# Effect of ASP2151, a Herpesvirus Helicase-Primase Inhibitor, in a Guinea Pig Model of Genital Herpes

**DOI:** 10.3390/molecules16097210

**Published:** 2011-08-25

**Authors:** Kiyomitsu Katsumata, Koji Chono, Kenji Sudo, Yasuaki Shimizu, Toru Kontani, Hiroshi Suzuki

**Affiliations:** Drug Discovery Research, Astellas Pharma Inc., 21, Miyukigaoka, Tsukuba, Ibaraki, Japan; Email: kiyomitsu.katsumata@jp.astellas.com (K.K.); koji.chono@jp.astellas.com (K.C.); kenji.sudou@jp.astellas.com (K.S.); yasuaki.shimizu@jp.astellas.com (Y.S.); toru.kontani@jp.astellas.com (T.K.)

**Keywords:** ASP2151, amenamevir, antiviral, HSV, guinea pig, genital herpes

## Abstract

ASP2151 is a herpesvirus helicase-primase inhibitor with antiviral activity against varicella zoster virus and herpes simplex virus types 1 (HSV-1) and 2 (HSV-2). Here, we examined the potency and efficacy of ASP2151 against HSV *in vitro* and *in vivo*. We found that ASP2151 was more potent in inhibiting the replication of HSV-1 and HSV-2 in Vero cells in the plaque reduction assay and had greater anti-HSV activity in a guinea pig model of genital herpes than did acyclovir and valacyclovir (VACV), respectively. Oral ASP2151 given from the day of infection reduced peak and overall disease scores in a dose-dependent manner, resulting in complete prevention of symptoms at the dose of 30 mg/kg. The 50% effective dose (ED_50_) values for ASP2151 and VACV were 0.37 and 68 mg/kg, respectively, indicating that ASP2151 was 184-fold more potent than VACV. When ASP2151 was administered after the onset of symptoms, the disease course of genital herpes was suppressed more effectively than by VACV, with a significant reduction in disease score observed one day after starting ASP2151 at 30 mg/kg, whereas the therapeutic effect of VACV was only evident three days after treatment at the highest dose tested (300 mg/kg). This indicated that ASP2151 possesses a faster onset of action and wider therapeutic time window than VACV. Further, virus shedding from the genital mucosa was significantly reduced with ASP2151 at 10 and 30 mg/kg but not with VACV, even at 300 mg/kg. Taken together, our present findings demonstrated the superior potency and efficacy of ASP2151 against HSV.

## 1. Introduction

Herpes simplex virus types 1 (HSV-1) and HSV-2 are prevalent pathogens belonging to the human herpesvirus family viruses that causes a variety type of human diseases. Genital infection with HSV-1 and HSV-2 results in genital herpes with vesicles or small, grouped ulcers in the genital region. After the primary infection HSV establishes latent infection in the sensory ganglia, followed by recurrent episodes of reactivation [1]. Viral subtypes differ in epidemiology, natural history and propensity for recurrence [2,3]; for instance, HSV-1 genital infections are typically milder and less prone to recurrence than HSV-2 [4,5,6,7,8], while HSV-2 causes genital herpes more frequently than HSV-1 [9,10]. While genital herpes cannot be cured at present, medications are available to treat episodes and minimize the symptoms. Since the late 1970s, synthetic nucleoside analogues targeting viral DNA polymerase, such as acyclovir (ACV), penciclovir, valacyclovir (VACV), and famciclovir, have been developed for use in treating HSV infections [11,12]. Although commonly used to treat primary and recurrent herpes virus infections, these compounds are not satisfactory in preventing the genital herpes, and their efficacy is reduced if administration is delayed after the appearance of symptoms [13,14,15]. Since those current drugs are all based on the same mechanism of action, emergence of cross-resistant HSV is one of potential concerns, and there is therefore a need to investigate novel anti-HSV agents.

**Figure 1:**
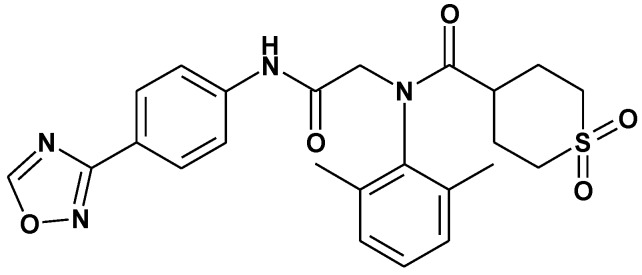
Molecular structure of ASP2151.

ASP2151 (Figure 1), an oxadiazolylphenyl derivative, is a novel type of anti-herpes agent targeting the herpesvirus helicase-primase complex, which consists of gene products essential for virus replication [16]. Two other classes of helicase-primase inhibitors (HPIs) with anti-HSV activity have been discovered: BILS 179 BS, an aminothiazolylphenyl-containing compound, and BAY 57-1293, a thiazole urea derivative [17,18]. These HPIs were reported to be specific inhibitors against HSV replication and possess *in vivo* antiviral activity in mouse and guinea pig models [17,18,19,20], demonstrating their potential as a new class of anti-HSV drugs. In contrast, ASP2151 possesses antiviral viral activity not only against HSVs, but also varicella-zoster virus, and the *in vivo* effect of ASP2151 on a HSV-1-infected zosteriform spread model was assessed in mice [16].

HSV-2 infection in guinea pigs is a well-established and predictive model for genital herpes in humans [21,22]. Following primary intravaginal infection with HSV-2, guinea pigs develop vesicular lesions with a disease course resembling that seen in humans [21,23,24,25,26,27]. The model has been widely used to study the pathogenesis of the disease and the influence of treatment on disease outcome. We, therefore, studied the anti-HSV activity of ASP2151 in the guinea pig model of human genital herpes.

## 2. Results and Discussion

### 2.1. Antiviral Activity of ASP2151 against HSV-1 and HSV-2

Using the plaque reduction method, the anti-HSV activity of ASP2151 compared with ACV was evaluated against several strains of HSVs that included clinical isolates and an ACV-resistant mutant. Table 1 summarizes the EC_50_ and EC_90_ values of ASP2151 and ACV for the HSV strains. ASP2151 inhibited the replication of 11 ACV-susceptible clinical isolates as well as three laboratory stock strains. The EC_50_ and EC_90_ values of ASP2151 ranged from 0.008 to 0.036 μM and from 0.026 to 0.12 μM, respectively. ASP2151 was also active against the ACV-resistant strain A4-3, an EC_50_ value of 0.026 μM, which showed reduced susceptibility to ACV (EC_50_ value, 49 μM). 

**Table 1:** Anti-HSV-1 and -2 activity and cytotoxicity of ASP2151 and ACV in Vero cells.

Virus strain	ASP2151		ACV	
	EC_50_ (μM) ^a^	EC_90_ (μM) ^a^	EC_50_ (μM) ^a^	EC_90_ (μM) ^a^
**HSV-1**				
*Laboratory stock*				
KOS	0.027 ± 0.001 *	0.062 ± 0.003 *	2.1 ± 0.2	7.0 ± 0.7
Miyama	0.022 ± 0.001 *	0.058 ± 0.002 *	1.7 ± 0.2	6.3 ± 0.7
*Clinical isolates from Japan*				
WT-51	0.030 ± 0.001 *	0.075 ± 0.003 *	0.73 ± 0.02	1.4 ± 0.1
H-5	0.028 ± 0.001 *	0.087 ± 0.007 *	1.4 ± 0.1	5.3 ± 0.5
Miyoshi	0.036 ± 0.001 *	0.087 ± 0.004 *	1.5 ± 0.1	5.5 ± 0.2
Fujito	0.029 ± 0.001*	0.065 ± 0.008 *	1.0 ± 0.2	2.6 ± 0.4
Endo	0.023 ± 0.001 *	0.064 ± 0.003 *	1.2 ± 0.1	3.2 ± 0.2
A4-3 (ACV-resistant)	0.026 ± 0.001 *	0.055 ± 0.006 *	49 ± 4	200 ± 11
*Clinical isolates from the USA*				
CI-25	0.009 ± 0.001 *	0.035 ± 0.002 *	0.82 ± 0.16	5.6 ± 0.8
CI-114	0.008 ± 0.001 *	0.026 ± 0.003 *	0.97 ± 0.05	3.6 ± 0.1
CI-116	0.010 ± 0.002 *	0.034 ± 0.002 *	0.95 ± 0.07	4.0 ± 0.4
**HSV-2**				
*Laboratory stock*				
G	0.025 ± 0.002 *	0.075 ± 0.005 *	1.6 ± 0.2	7.3 ± 0.9
Lyon	0.034 ± 0.002 *	0.12 ± 0.01 *	2.6 ± 0.1	8.0 ± 0.3
*Clinical isolate from Japan*				
Kondo	0.023 ± 0.001 *	0.064 ± 0.003 *	1.4 ± 0.1	4.3 ± 0.5
*Clinical isolates from the USA*				
CI-27	0.015 ± 0.002 *	0.052 ± 0.007 *	3.5 ± 0.4	19 ± 2
CI-5243	0.014 ± 0.003 *	0.057 ± 0.009 *	1.1 ± 0.2	6.0 ± 0.6
Cytotoxicity, CC_50_ (μM) ^b^	>200		>200	
SI, CC_50_/EC_50_^c^	>5500		>57	

^a^ Antiviral activity (EC_50_ and EC_90_) was determined using the plaque reduction assay. The data represent the mean ± SE of four independent experiments using each strain; ^b^ Data represent the mean of three independent experiments. Values for CC_50_were determined using Neutral Red assay in proliferating Vero cells; ^c^ SI represents the smallest value among ACV-susceptible strains tested. * *P * < 0.05 as compared with ASP2151 and ACV (Student’s t-test).

Further, the EC_50_ and EC_90_ values of ASP2151 against all HSV strains were statistically significantly lower than those of ACV. ASP2151 showed no obvious cytotoxic effects in Vero cells even at relatively high concentrations (CC_50_ value >200 μM), and thus the selectivity index (SI, CC_50_/EC_50_) was calculated to be at least 5,500 (Table 1).

### 2.2. Antiviral Efficacies of ASP2151 and VACV in HSV-2-Infected Guinea Pigs

**Figure 2:**
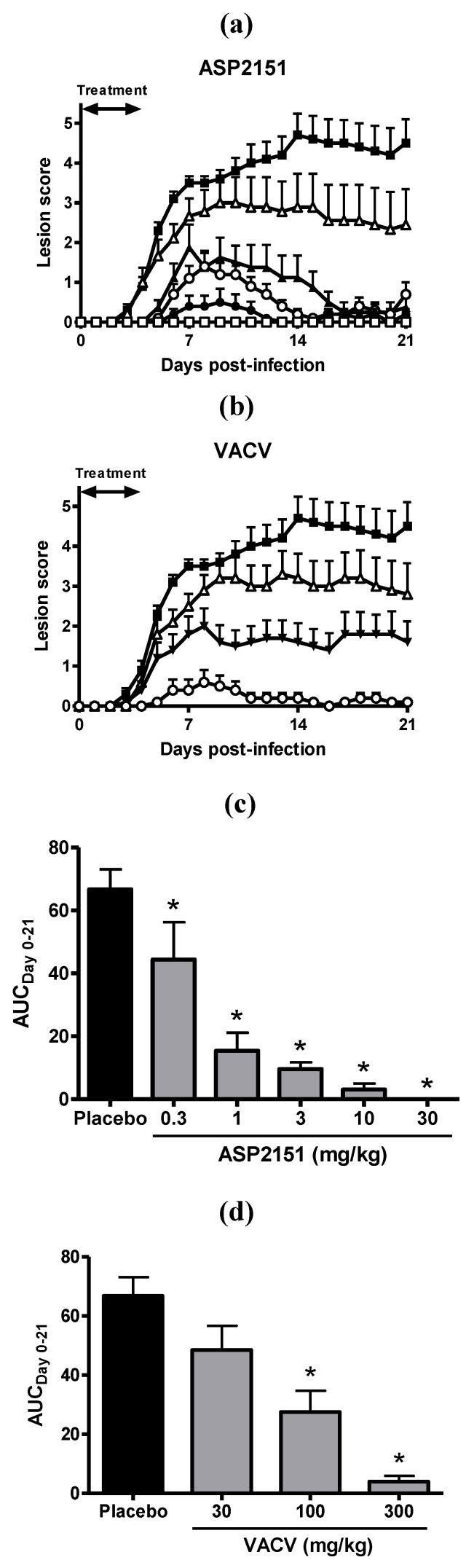
Efficacies of ASP2151 and VACV on genital herpes symptoms in a guinea pig model of HSV-2 infection. Guinea pigs vaginally infected with HSV-2 were orally administered placebo (closed square); ASP2151 at a dose of 0.3 (open triangle), 1 (closed triangle), 3 (open circle), 10 (closed circle), or 30 mg/kg (open square); or VACV at a dose of 30 (open triangle), 100 (closed triangle), or 300 mg/kg (open circle) twice daily from Days 0 to 4 post-infection. The mean disease scores for ASP2151- **(a)** or VACV-treated **(b)** groups were calculated and plotted against days post-infection; **(c)** and **(d)** represent the area under the disease score-time curve from Days 0 to 21 post-infection (AUC_Day0–21_; score × day). Data are expressed as the mean + SE of 8-10 animals per group. * *P * < 0.05, compared with the placebo group (Dunnett’s multiple comparison test).

The activity of ASP2151 against genital herpes infection was compared with VACV in a guinea pig model of HSV-2 infection. ASP2151 and VACV were orally administered twice daily for 5 days starting 3 h after HSV-2 strain G infection, and disease symptoms were monitored for 21 days. Results showed that ASP2151 dose-dependently reduced peak and overall disease scores with an ED_50_ of 0.37 mg/kg, while VACV reduced HSV pathogenicity with an ED_50_ of 68 mg/kg (Figure 2). These findings therefore indicate that ASP2151 was 184-fold more potent than VACV, based on ED_50_ values. 

Compared with placebo, oral administration of ASP2151 and VACV significantly reduced the cumulative disease score and AUC_Day0-21_ at doses of ≥0.3 and ≥100 mg/kg, respectively (*P* < 0.05). In addition, ASP2151 completely inhibited the development of disease symptoms at a dose of 30 mg/kg, while a 10-fold higher dose of VACV (300 mg/kg) did not completely suppress the symptoms.

### 2.3. Therapeutic Efficacies of ASP2151 and VACV on Genital Herpes Symptoms in HSV-2-Infected Guinea Pigs

**Figure 3:**
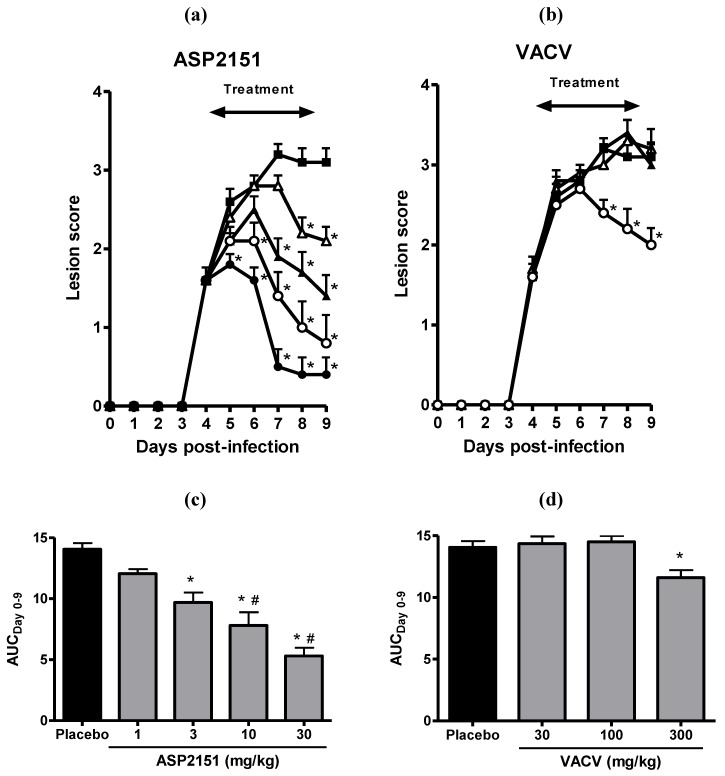
Therapeutic efficacies of ASP2151 and VACV on genital herpes symptoms in a guinea pig model of HSV-2 infection. Guinea pigs vaginally infected with HSV-2 were orally administered placebo (closed square); ASP2151 at a dose of 1 (open triangle), 3 (closed triangle), 10 (open circle), or 30 mg/kg (closed circle); or VACV at a dose of 30 (open triangle), 100 (closed triangle), or 300 mg/kg (open circle) twice daily from Days 4 to 9 post-infection. The mean disease scores for each ASP2151- **(a)** or VACV-treated **(b)** groups were calculated and plotted against days post-infection. * *P* < 0.05, compared with the placebo group (Dunnett’s multiple comparison test); **(c)** and **(d)** represent the area under the disease score-time curve from Days 0 to 9 post-infection. Data are expressed as the mean + SE of 10 animals per group. * *P* < 0.05, compared with the placebo group (Dunnett’s multiple comparison test). ^#^ *P* < 0.05, compared with the AUCs of valaciclovir 300 mg/kg treatment group (Tukey’s multiple comparison test).

Treatment with ASP2151 and VACV was initiated at Day 4 post-infection only in animals with genital herpes vesicles in order to replicate the clinical situation whereby patients present for medical attention after genital symptoms became apparent. Therapy was continued for a total of 5 days. ASP2151 administered twice daily showed a dose-dependent reduction in peak and overall disease scores, resulting in significant inhibition of genital herpes pathology at doses of 3, 10, and 30 mg/kg as measured by the AUC of the mean disease scores compared with the placebo group (*P * < 0.05) (Figure 3). VACV also significantly reduced the AUC at 300 mg/kg but not at 30 or 100 mg/kg (Figure 3). The AUCs at doses of 10 and 30 mg/kg ASP2151 were statistically significantly smaller than that of VACV at 300 mg/kg. Further, the progression of disease symptoms was markedly abrogated in the HSV-2-infected guinea pigs treated with ASP2151 (Figure 3). The significant reduction in disease score was already observed on Days 5 and 6 post-infection in guinea pigs treated with ASP2151 at 30 mg/kg and 10 mg/kg, respectively, whereas the therapeutic effect of VACV at 300 mg/kg was first evident on Day 7 post-infection.

### 2.4. Therapeutic Efficacies of ASP2151 and VACV on Viral Shedding

**Figure 4:**
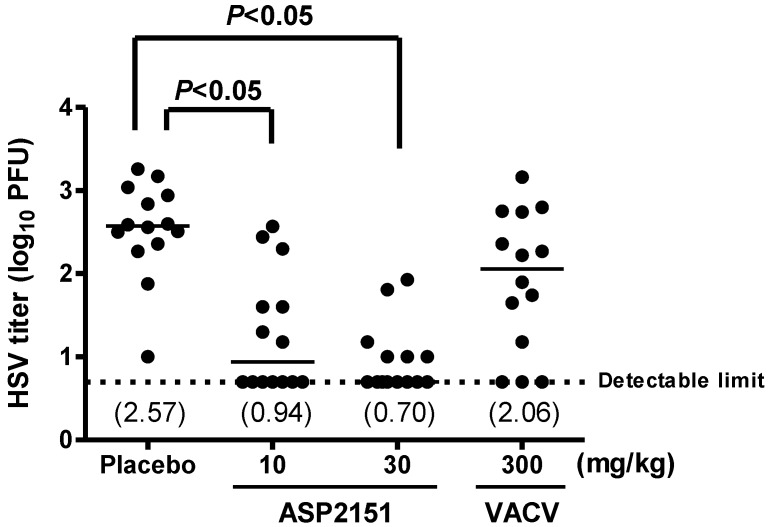
Efficacies of ASP2151 and VACV on genital titers on Day 5 post-infection in a guinea pig model of HSV-2 infection. Each datum point represents the HSV-2 titer for each animal in the respective treatment groups (n = 14). The horizontal line of each group marks the median HSV-2 titer level with the viral titers indicated in parentheses. The horizontal dotted line represents the detection limit of the assay. *P * < 0.05, comparison with placebo was statistically analyzed using Steel’s multiple comparison test.

To investigate the effect of ASP2151 on HSV shedding from the genital mucosa, we measured virus titers with swabbing samples of vaginal secretions obtained on Days 5 and 8 post-infection in animals treated with ASP2151 and VACV. The HSV titers on Day 4 post-infection are presented in Figure 4. The infected placebo group exhibited titers ranging from 1.00 to 3.26 log_10_ PFU, with a median log_10_ PFU of 2.57 on Day 5 post-infection. At doses of 10 or 30 mg/kg ASP2151, respectively, seven and eight out of 14 animals had viral titers below the detection limit. In contrast, treatment with VACV twice daily at 300 mg/kg did not significantly reduce the virus content in the genital swab on Day 5 post-infection. On Day 8 post-infection, 11 out of 14 animals in the placebo group and all animals in the ASP2151- and VACV-treated groups had viral titers below the detection limit (data not shown).

### 2.5. Discussion

In the present study, we examined the effect of ASP2151 on HSV replication both *in vitro* and *in vivo*. *In vitro* plaque reduction assay revealed that ASP2151 efficiently inhibited viral replication of HSV-1 and HSV-2, with significantly potent activity compared to ACV and no obvious cytotoxicity. Potent antiviral activity of ASP2151 was also observed in female guinea pigs vaginally infected with HSV-2. In the genital herpes model, ASP2151 prevented development of genital symptoms under treatment from the day of infection with lower effective doses than VACV, also suppressed the disease course more rapidly than VACV when initiation of treatment was delayed, and significantly decreased genital tract HSV shedding. Our findings thus revealed that ASP2151 effectively inhibited the genital symptoms with potent antiviral activity against HSV-2 replication in guinea pigs.

The guinea pig model of genital herpes is suggested to be clinically relevant to human situation and suitable to evaluate the antiviral effects of novel candidates [18,28,29,30,31,32,33]. Although HSV-2 is used in the guinea pig model in most previous reports and in the present study, it is known that HSV-1 also causes genital herpes [34]; we therefore deemed it important to assess the antiviral spectrum of ASP2151 using both HSV strains. ASP2151 showed similar potent *in vitro* antiviral activity against both HSV-1 and HSV-2, with significantly lower EC_50_ values compared to ACV, and the activity against each HSV strain was comparable to that seen in the HSV-2 strain G used in the *in vivo* studies (Table 1). It is therefore anticipated that the antiviral spectrum and efficacy of ASP2151 satisfy the requirements for treating genital herpes.

To more accurately reflect the clinical situation whereby patients seek medical attention after the appearance of genital symptoms, we initiated treatment after the appearance of herpes symptoms in the animal model to study the therapeutic efficacy of ASP2151 and VACV. The progression of disease symptoms was markedly abrogated in HSV-2-infected guinea pigs treated with ASP2151, with a significant reduction in disease score after Days 5 and 7 post-infection in guinea pigs treated with ASP2151 and VACV, respectively (Figure 2). These results indicate the superior therapeutic efficacy of ASP2151 compared with VACV with an earlier relief of symptoms in the HSV-2-infected guinea pig model. The other HPIs, BAY 57-1293 and BILS 179 BS, also exhibited greater efficacy than VACV or ACV in a delayed therapy regimen [17,18]. ACV had little [35,36] or no effect [37] when commenced 72 h post-infection in guinea pigs; further, patients experience clinical benefit only when oral therapy is initiated early—usually within 24 h of onset of the disease [14,15]. Therefore, the broad therapeutic time window of ASP2151 may be a significant advantage over other agents.

HSV-infected individuals exhibit spontaneous viral shedding regardless of the presence of recurrent active disease; as such, HSV transmission is a concern for genital herpes patients and their partners. VACV is approved for use in reducing the transmission of genital herpes. Suppressive treatment with VACV prevented transmission of genital herpes in discordant couples in a placebo-controlled trial [38], suggesting that prevention of HSV replication may lead to a reduction of the risk of transmission. Although ACV reduces virus shedding in the genital tract, the virus can still be detected by DNA polymerase chain reaction during suppressive therapy [39]. VACV and ACV are highly effective in suppressing the frequency and quantity of HSV shedding, but are unable to totally cease shedding [40]. In the guinea pig model of HSV-2 genital infection, viral titers in vaginal swabs were not altered following treatment with ACV and VACV [19,41]. In the present study, we found that ASP2151 significantly reduced virus shedding, whereas no obvious reduction was observed in the VACV treatment group (Figure 4), a finding consistent with other HPI studies showing a prominent effect on virus shedding with the use of BAY 57-1293 and BILS 179 BS, [17,18], leading to suppression of genital herpes recurrence in guinea pigs [18,19]. Extrapolating from animal experiments, we expect ASP2151 to show a similar suppressive effect on recurrence and transmission in humans.

Although VACV was clearly less potent or efficacious in the therapeutic regimen than ASP2151 in the guinea pigs, it is necessary to consider species specific phenomenon with regard to pharmacokinetics. Given that, unlike mice, dogs, or humans, guinea pigs are known to metabolize a significant proportion of VACV, we determined the plasma concentration of ACV, an active metabolite of VACV, in guinea pigs receiving VACV. We found that the plasma concentration of ACV increased in proportion to the dose after a single oral dose of 30, 100, or 300 mg/kg VACV. The trough plasma ACV concentration in humans receiving 500 mg of VACV twice daily (an approved treatment regimen for recurrent genital herpes) was exceeded only in the 300 mg/kg VACV treatment group (data not shown). In addition, the 300 mg/kg dose of VACV was the maximum dose possible in this animal model due to the adverse event signals (e.g., body weight loss) at higher dosing. These results suggested that ASP2151 may have superior or at least equivalent efficacy compared with VACV in a clinical setting. The clinical efficacy and tolerability of ASP2151 was recently confirmed in patients with recurrent genital herpes in Phase II clinical studies (manuscript in preparation).

## 3. Experimental Section

### 3.1. Drugs

ASP2151 (international nonproprietary name: amenamevir, Figure 1) was synthesized at Astellas Pharma Inc. (Tokyo, Japan). ACV (Sigma-Aldrich, St. Louis, MO, USA) and VACV (Valtrex® film tablets; GlaxoSmithKline, Middlesex, UK) were purchased from commercial suppliers.

### 3.2. Viruses and Cell Lines

HSV strains clinically isolated in the US were kindly provided by Dr. Nancy Sawtell (Cincinnati Children's Hospital Medical Center, Cincinnati, OH, USA). Other viruses and cell lines were provided by Rational Drug Design Laboratories (Fukushima, Japan). Human embryonic fibroblast (HEF) cells [42] and Vero cells were grown in Eagle’s minimum essential medium supplemented with 10% fetal bovine serum (FBS), 100 units/mL penicillin G, and 100 μg/mL streptomycin (Invitrogen, Carlsbad, CA, USA). HSV-1 and HSV-2 were propagated using HEF cells in maintenance medium containing 2% FBS.

### 3.3. Plaque Reduction and Cytotoxicity Assays

The antiviral activity of ASP2151 and ACV against HSV was tested using a plaque reduction assay as described previously [16]. Briefly, Vero cells were seeded into 24-well tissue culture plates at 1 × 10^5^cells/well and incubated until the cells formed a monolayer. After the medium was removed, the cells were infected with HSV-1 or HSV-2 at a titer of 40 plaque-forming units (PFU)/well. The plates were then incubated for 1 h at 37 °C. After two washes with maintenance medium, cells were treated with the test compound until clear plaques appeared, after which they were then fixed with 10% formalin in phosphate-buffered saline and stained with 0.02% Crystal Violet solution. The number of plaques was then counted under a microscope. The 50% effective concentration (EC_50_) and the EC_90_—the concentrations that reduce the plaque numbers by 50% and 90%, respectively—were calculated using nonlinear regression analysis with a sigmoid-E_max_ model. Neutral Red assay was conducted using Vero cells to determine the cytotoxic concentration that causes a 50% reduction in the number of viable cells (CC_50_).

### 3.4. *In Vivo* Antiviral Activity

All animal experimental procedures were approved by the Animal Ethical Committee of Yamanouchi Pharmaceutical Co., Ltd. (now known as Astellas Pharma Inc). Female guinea pigs (Hartley, aged 4 weeks at the time of viral infection) were intravaginally infected (designated as Day 0 post-infection) with a cotton swab saturated with PBS containing HSV-2 strain G as described previously [34]. For HSV-2 strain G, the virus pool contained 1.25 × 10^5^ PFU/mL and caused lesions in nearly 100% of control animals. ASP2151 at 0.3, 1, 3, 10, or 30 mg/kg, or VACV at 30, 100, or 300 mg/kg (suspension in 0.5% methylcellulose solution), was orally administered twice daily for 5 days starting 3 h after viral inoculation as a prophylactic treatment, or 4 days after viral inoculation as a therapeutic treatment. 

The disease course was monitored daily for 21 days and scored on a 0-6 composite scale based on the severity of vaginitis and neurological symptoms according to the following criteria: score 0, no signs of infection; score 1, localized, barely perceptible, small vesicles; score 2, small or large vesicles involving 10% to 50% of the area; score 3, small or large vesicles involving 50% to 100% of the area; score 4, small ulcers involving 10% to 50% of the area; score 5, severe ulcers involving 50% to 100% of the area; and score 6, hind limb paralysis or death. Disease extent was measured from the area under the curve (AUC) of the mean daily disease score each day after viral inoculation. The effect of treatment was based on a reduction in AUC of the disease curve in the presence of ASP2151 or VACV compared with vehicle alone. ED_50_ values for ASP2151 and VACV were calculated from dose-inhibition curves using linear regression analysis of the linear portion of the curves. Potencies of ASP2151 and VACV were then compared using ED_50_ values after the parallelism test was performed.

### 3.5. Virus Detection

Swab samples of vaginal secretions were collected with a pre-moistened rayon swab. The swab samples were placed in 1 mL of maintenance medium and stored frozen at −80 °C until virus titration using the plaque assay. Vero cells were seeded into a 24-well tissue culture plate at 1 × 10^5^ cells/well and incubated at 37 °C in a humidified atmosphere with 5% CO_2_ for 3 days. After confirming that the cells had formed a monolayer, the growth medium was removed, and cells were infected with serial 10-fold dilution of the swab specimens. After subsequent centrifugation at 1500 rpm for 40 min at room temperature, the plate was incubated for 1 h at 37 °C and washed three times with maintenance medium. After maintenance medium containing 0.8% methylcellulose was added, the plates were incubated for 3 days at 37 °C in a 5% CO_2_ incubator. Cell monolayers were fixed in 10% formalin/ phosphate-buffered saline, stained with 0.02% crystal violet, and the number of HSV-2 plaques was counted under a microscope. The HSV-2 titers in the vaginal secretions were calculated as log_10_ PFU. The lower limit of detection for this assay was 0.7 log_10_ PFU.

### 3.6. Statistical Analyses

In all cases, statistical analyses were performed using SAS software (SAS Institute, Cary, NC, USA), and *P * < 0.05 was considered statistically significant. In the plaque reduction assay, results were analyzed using Student’s t-test for comparisons between ASP2151 and ACV. The prophylactic effects of ASP2151 and VACV (treatment started from the day of infection) were analyzed in terms of reduction in AUC_Day0–21_ of the disease score compared with placebo using Dunnett’s multiple comparison test. The therapeutic effects of delayed treatment with ASP2151 and VACV were first analyzed in terms of reduction in AUC_Day0–9_ of the disease score compared with placebo using Dunnett’s multiple comparison test. To compare the therapeutic efficacy of ASP2151 with VACV, the data were analyzed again via Tukey’s multiple comparison test. Changes in scores over time were assessed by repeated-measurement two-way ANOVA, and when interactions were considered significant, data were assessed using Dunnett’s multiple comparison test at each time point of measurement.

With regard to HSV-2 titers in swab samples, Dunnett’s multiple comparison test was used to compare lesion scores between the placebo and the treatment groups on Days 5 and 8 post-infection. The two-tailed Steel’s test was used to compare genital HSV-2 titers on Day 5 after infection between the placebo and treatment groups.

## 4. Conclusions

In conclusion, we showed that in a guinea pig model of genital herpes ASP2151 is superior to VACV in both potency and efficacy. Based on our study, ASP2151 merits further investigation with regard to its use in treating HSV-1 and HSV-2 infections.
